# Epidemiology and Clinical Characteristics of Acute Plant Exposure in Patients Aged between 0 and 18 Years—A Six-Year Retrospective Study

**DOI:** 10.3390/children11030271

**Published:** 2024-02-21

**Authors:** Gabriela Viorela Nițescu, Andreea Grama, Teodora Turcu, Andreea Strătulă, Ana Dragomirescu, Elena Silvia Pană, Andreea Baciu, Daniela Luiza Baconi, Maria Dorina Crăciun, Coriolan Emil Ulmeanu

**Affiliations:** 1 Pediatric Poison Centre, “Grigore Alexandrescu” Clinical Emergency Hospital for Children, 017443 Bucharest, Romania; rodica-andreea.grama@umfcd.ro (A.G.); teodora-adela.turcu@drd.umfcd.ro (T.T.); andreea.stratula@stud.umfcd.ro (A.S.); ana.dragomirescu@rez.umfcd.ro (A.D.); elena-silvia.pana@rez.umfcd.ro (E.S.P.); andreea.baciu@rez.umfcd.ro (A.B.); maria.craciun@umfcd.ro (M.D.C.); coriolan.ulmeanu@umfcd.ro (C.E.U.); 2 Department of Pediatrics, Faculty of Dentistry, “Carol Davila” University of Medicine and Pharmacy, 020021 Bucharest, Romania; 3 Department of Toxicology, Faculty of Pharmacy, “Carol Davila” University of Medicine and Pharmacy, 020021 Bucharest, Romania; daniela.baconi@umfcd.ro; 4 Department of Epidemiology, Faculty of Medicine, “Carol Davila” University of Medicine and Pharmacy, 020021 Bucharest, Romania

**Keywords:** plant, poisoning, children, adolescents

## Abstract

Background: Exposure to plants accounts for approximately 5% of human poisoning cases reported by poison control centers in North America and Europe. The aim of this study was to investigate acute plant poisoning in patients aged 0–18 years admitted to a Romanian pediatric poison center, focusing on epidemiological and clinical aspects. Methods: A retrospective observational study was conducted between 2017 and 2022, analyzing medical records for demographic information, clinical features, biological findings, and outcomes. Statistical analysis was performed using Microsoft Excel. Results: 71 patients (aged 7 months to 16 years) presented with acute plant poisoning. Most cases were unintentional (92.9%), peaking during the autumn season. *Colocasia* (18.3%), *Dieffenbachia* (9.8%), and *Ricinus* (5%) were the most frequently involved plants. Gastrointestinal symptoms, especially vomiting, predominated. The Poisoning Severity Score classified most cases as mild (52.1%), with no severe or fatal cases. The mean length of hospitalization was 1.8 days. Conclusions: Unintentional plant exposure, mainly in children under 5 years of age, accounted for more than 90% of cases. Gastrointestinal exposure and symptoms were prevalent, and treatment consisted mainly of symptomatic and supportive measures. Severe and fatal cases were rare, highlighting the generally favorable outcome and low incidence of severe poisoning in the pediatric population.

## 1. Introduction

Plant exposure is one of the most common types of human poisoning, accounting for about 5%, as shown in the reports of various poison control centers in North America and Europe [1]. The large number of exposure cases occurring in the pediatric population is probably due to the accessibility and attractiveness of plants to this age group. The majority of cases in young children are produced by acute ingestion, but there are also reports of adolescents consuming plants for recreational or self-injury purposes. Data published by the American Association of Poison Control Centers and by European Association of Poisons Centres and Clinical Toxicologists showed that the majority of exposed patients were asymptomatic, fewer than 20% had minor to moderate symptoms, and very few necessitated serious interventions [2,3]. However, in other parts of the world where health facilities are less accessible, plant exposure, particularly when used for self-injury, can pose a significant risk [4,5].

Eastern Europe, including Romania, remains a strongly represented area with a diverse array of plants. The wide availability of domestic and ornamental plants in households predisposes people to accidental poisoning, especially children. These cases of unintentional poisoning are a significant health hazard, causing occasionally severe toxic effects in children [6]. The severity of symptoms ranges from mild and moderate—oral cavity and pharynx soreness, eye irritation, high fever or hypothermia, profuse cold perspiration, deglutition difficulties, nausea, vomiting, headache, diarrhea, numbness, weakness, paresthesia, peripheral tremor, and equilibrium disorders—to severe and fatal—hepatic and renal failure, coma, and cardio-respiratory arrest [7,8].

Toxicity varies depending on the part of the plant (leaves, stem, root, seeds, or flowers), the age of the plant (whether freshly harvested or pre-dried), the method of preparation (for homeopathic remedies—syrups, tinctures, and lotions), and the exposure route (dermal, oral, or via inhalation) [9]. An important role is also played by their composition, which is very complex, with each plant containing multiple xenobiotics that work independently or in concert.

Treatment is generally symptomatic and supportive in cases of acute poisoning, with most requiring only a short period of hospitalization without further major complications and the need for follow-up. Occasionally, severe allergic reactions (e.g., Quincke’s edema or anaphylactic shock) require urgent medical intervention with specific therapy as they have a high mortality rate [10]. In certain instances, primary decontamination procedures are paramount, encompassing interventions such as the administration of activated charcoal and gastric lavage, or alternatively, endoscopic extraction of botanical matter from the gastric cavity. Administering activated charcoal is recommended within the initial 30–60 min following ingestion and is particularly indicated in instances characterized by moderate intoxication [11]. However, it is contraindicated in patients exhibiting coma, non-cooperation, or recurrent emesis. In cases of severe toxicity, resorting to secondary decontamination via hemodialysis may be deemed appropriate [12]. The application of specific antidotes remains confined to a limited subset of plants, including the utilization of physostigmine for alkaloid-induced toxicity or antidigoxin antibodies for cardiac glycoside-related poisoning. It is important to note that the administration of antidotes does not guarantee immediate recovery, as vigilant and sustained monitoring, alongside an array of paraclinical assessments, are necessary to obtain a favorable outcome [13].

One of the most effective ways of minimizing accidental poisoning is raising awareness of the toxicity of plants and their harmful effects. Accurate information and appropriate education on the toxicity of plants should be made available to the scientific community, the general public, educators, and public authorities, who together can play a significant role in this issue [3,14,15].

The aim of this study is to present the epidemiologic and demographic characteristics of pediatric patients with acute plant poisoning, to identify the main plant species involved in a part of the Central and Eastern European region, to describe the clinical picture and paraclinical changes according to the plant species, and to evaluate the outcome of the patients.

## 2. Materials and Methods

A descriptive retrospective observational study was conducted to investigate the epidemiology, clinical features, and outcomes of acute plant exposure in patients aged 0–18 years admitted to the Toxicology Clinic of “Grigore Alexandrescu” Clinical Emergency Hospital for Children, Bucharest, over a 6-year period, from 2017 to 2022.

This study included cases diagnosed with acute plant exposure with the T62.2 ICD-10-AM diagnosis code. We excluded from this study all cases of acute exposure to the following: mushrooms, phytomedicines (industrially processed plant materials like tinctures, mixtures, and teas), industrial botanical ingredients and foodstuffs, tobacco (*Nicotiana tabacum*), and *Cannabis sativa*.

The medical records and internal hospital database were reviewed to collect epidemiological and demographic data (such as age, sex, and area of occurrence), year and month of admission, length of hospitalization, plant species and clinical features (signs and symptoms on admission and complications), biological parameters, clinical outcome, and treatment course for every patient.

In order to correctly identify the type of plant, we used the part of the plant that caregivers brought to the hospital, photographs, and descriptions correlated with botanical images from the aforementioned sources.

The severity of the cases of poisoning was assessed using the Poisoning Severity Score (PSS) developed by the European Association of Poison Centers and Clinical Toxicologists, the World Health Organisation International Program on Chemical Safety (IPCS), and the European Commission. Poisoning Severity Scores (PSSs) grade poisoning severity based on signs and symptoms as follows: PSS = 0 (none), PSS = 1 (mild), PSS = 2 (moderate), PSS = 3 (severe), PSS = 4 (fatal) [16].

Data were processed and analyzed using Microsoft Excel 2023 (Microsoft Corporation, Redmond, WA, USA), and descriptive statistics such as the mean, median, and percentage were calculated as appropriate for each type of data.

This study was conducted in accordance with the Declaration of Helsinki and was approved by the Ethics Committee Decision registered under the number 19234/30 June 2023. Informed consent was obtained from each patient’s guardian, according to our existing Hospital Policy. This policy means that for all inpatients, informed consent is required for inclusion in any study conducted in the hospital.

## 3. Results

**Figure 1:**
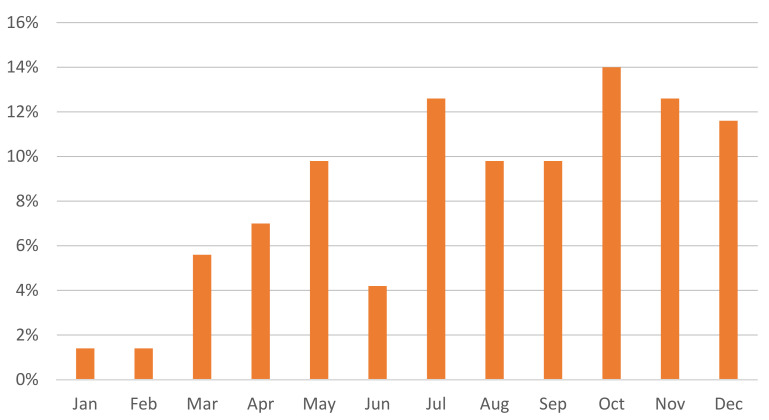
Distribution of acute plant exposure cases by month—most cases occurred in the autumn months.

In total, 71 cases of acute plant poisoning were identified during the 6-year study period, from 2017 to 2022. All of the patients (*n* = 71) were admitted to the hospital for observation and treatment. The distribution of cases by month showed that most of them occurred during the autumn months (26, 36.6%), followed by summer (19, 26.7%), spring (16, 22.5%), and winter (10, 14.1%), as detailed in Figure 1.

### 3.1. Demographic Data

The mean age of the patients was 4.6 years, ranging from 7 months to 16 years. Of all the cases, 32 patients (45.0%) were male and 39 (54.9%) were female, resulting in a male-to-female ratio of 1:1.2.

Analyzing the place of occurrence, we noticed that 40 (56.3%) of the acute poisoning cases were reported in urban areas and 31 (43.6%) in rural areas, as detailed in Table 1.

### 3.2. Intention

Concerning intention, data analysis showed that most cases of poisoning were unintentional (66 cases, 92.9%), while in four cases (5.3%), an intentional reason was observed. The intention could not be determined in one case (1.4%). All cases of poisoning in the study group were caused by ingestion or contact between the plant and the oral cavity.

### 3.3. Etiology—Plant Species

The plant was successfully identified in 69 (97.2%) of the cases. However, in two cases, the plant species could not be identified. A total of 30 species of plants were distinguished as causative agents. Domestic plants were involved in 36 (52.2%) cases and wild plants in 33 (47.8%) cases.

*Colocasia* (elephant’s ear) was the most identified plant, accounting for 13 (18.3%) cases, followed by *Dieffenbachia* 7 (9.9%), and *Ricinus* 5 (5%). *Atropa belladonna* and *Phytolacca decandra* were each identified in four cases (5.6%), followed by *Datura stramonium* and *Sambucus* spp. in three cases (4.2%) each. *Solanum capsicum*, *Viscum album, Hedera, Sambucus ebulus*, *Hevea brasiliensis*, *Nerium oleander*, *Taxus baccata*, and *Aconitum* were identified in two cases each (2.8%). The rarest findings, with only one case (1.4%) each, were *Solanum nigrum*, *Wisteria*, *Strychnos nux vomica*, *Syngonium podophyllum*, *Solanum dulcamara*, *Litchi chinensis*, *Spathiphyllum wallisii*, *Sambucus*, *Symphoricarpos albus*, *Hyacinthus orientalis*, *Laburnum anagyroides*, *Amaranthus caudatus*, *Arum alpinum*, *Conium maculatum*, *Zamioculcas zamiifolia*, *Platanus*, and *Pyracantha coccinea*. Five (16.6%) of the plant species that were identified in our study group are classified as highly toxic plants, namely, *Datura stramonium*, *Atropa belladonna*, *Dieffenbachia*, *Ricinus*, and *Aconitum*.

### 3.4. Clinical Features

The clinical picture revealed a variety of presentation forms, matching the diversity of the etiology, with a predominance of gastrointestinal symptoms, including oral cavity injuries (21.2%), sialorrhea (10.6%), vomiting (14.6%), abdominal pain (4%), and nausea (5.3%). Acute dehydration occurred in 22% of the patients. The clinical features consistent with etiology are illustrated in Table 2.

### 3.5. Biological Findings

The biological findings included elevated transaminases in 14.1% of the patients, hyperglycemia in 26.8% of the cases, and hypoglycemia in 1.4%. Additionally, five patients (7.1%) presented elevated blood urea levels, and three patients (4.2%) had elevated blood creatinine. Metabolic acidosis was observed in 4.2% of the cases and metabolic alkalosis in 2.8% of them.

### 3.6. Severity

The Poisoning Severity Score (PSS) was used to assess the severity of the clinical picture in the study population and was applied in all cases. The distribution of PSS was as follows: PSS = 0 in 20 (28.2%) cases, PSS = 1 in 37 (52.1%) cases, PSS = 2 in 14 (19.7%), and no cases of severe or fatal poisoning (PSS = 3 or PSS = 4) were identified in our study. The severity of poisoning in relation to plant species is illustrated in Table 2.

### 3.7. Treatment and Clinical Outcome

Gastric decontamination was performed in 5 cases (7.1%), water–electrolyte re-equilibration with intravenous fluids was necessary in 58 cases (81.7%), antihistaminic treatment was administered to 21 patients (29.5%), and corticosteroid treatment was required in 5 cases (7.1%). In terms of symptomatic treatment, six patients (8.5%) received antiemetics, and antiacids were recommended in five cases (7.1%).

All of the patients were discharged without sequelae, 65 (91.5%) of them being hospitalized for 2 days or less. The length of hospitalization ranged from 1 to 4 days, with a mean of 1.8 days and a median of 2 days.

## 4. Discussion

The findings of this retrospective study, conducted in a pediatric toxicology unit over a 6-year period, contribute to the existing body of knowledge regarding acute plant exposure, highlighting epidemiological and clinical characteristics in patients aged 0–18 years.

Our study identified 71 cases of acute plant exposure. The mean age of the patients was 4.66 years, ranging from 7 months to 16 years, slightly higher than was reported in previous studies, which show values between 1 and 3 years [9,16,17]. The male-to-female ratio was 1:1.2. This slightly higher prevalence in females is not usually found in similar studies, which show a higher prevalence in males [5,9,17].

A higher incidence of exposure occurred in urban areas. This may be due to the greater availability of domestic and ornamental plants in urban areas, predisposing children to accidental exposure. Another explanation could be the greater availability and accessibility of health facilities in these areas, leading to better reporting and documentation of cases. It is important to note that the reasons for the higher prevalence of plant exposure in urban areas are likely to be complex and multifactorial and may vary depending on the specific location and context. Limited information on this epidemiological aspect was found in the current medical literature.

Interestingly, the incidence of acute plant exposure decreased over the study period, with the highest number of cases occurring in 2017 (30.98%) and the lowest in 2021 and 2022 (11.26%). This may be due to COVID-19 pandemic restrictions implemented during this period, which probably led to either limited access to hospitals or better surveillance of children at home.

Seasonal variations in plant exposure have been consistently documented in the literature, with most authors showing that these tend to peak during the months of August and September [18,19].

In our study, the highest number of cases occurred during the autumn months, followed by summer, then spring and winter. This could be explained by the types of plants involved, which are most common during different seasons, as well as children’s outdoor activities at certain times of the year. For instance, in autumn, there is an increase in cases related to plant berries and fruits that may be attractive to children. Similarly, in spring, there is an increase in cases of exposure related to newly emerging plants that may be mistaken for edible plants. Another reason for the seasonal variation in cases of plant exposure could be the behavior of children. In the study region, children tend to spend more time outdoors in autumn and summer (because of the holiday period and weather conditions). Consequently, they may come into contact with plants while playing. In contrast, children spend more time indoors during the winter, reducing their exposure to outdoor plants [19]. The majority of plant exposure cases in this study were unintentional, with ingestion or oral contact being the unique main route of exposure.

*Colocasia*, *Dieffenbachia*, and *Ricinus* were the most commonly identified plant species in our study.

*Colocasia* (*Colocasia esculenta* spp.), also known as “dasheen”, “taro”, or “elephant ear” for its large leaves of up to 40 cm in length, is a perennial domestic plant belonging to the Araceae family. The leaves are poisonous due to the presence of insoluble calcium oxalate crystals and the presence of needle-shaped raphides in the plant cells [1]. Leaf ingestion can cause lip, tongue, and pharyngeal edema, dysphagia, sialorrhea, nausea, vomiting, and diarrhea. In some severe cases, airway obstruction may occur due to significant swelling [20,21,22]. In our study, *Colocasia* was implicated in 13 cases (18.31%), with the noted symptoms being Quincke’s edema, hypersalivation, local hyperemia, and vomiting.

*Dieffenbachia* (Dumb cane), another member of the Araceae family, is an important cause of symptomatic plant exposure, particularly in the pediatric population [23]. It is characterized by strong stems, reaching 2–2.5 m. The leaves have a sheathing petiole and are either entirely green or are often variegated with irregular patterns of white or yellow [1]. Etiological data in our study ranked *Dieffenbachia* as the second most common cause of plant exposure. Its ingestion can cause severe mucous membrane irritation, due to local tissue damage caused by oxalate crystals present in the plant sap. Plant species containing insoluble oxalates exhibit a distinctive form of toxicity compared to those containing soluble oxalates. Insoluble oxalates, particularly in the form of calcium oxalate, assume the crystalline morphology of needle-shaped structures known as raphides [22]. In certain botanical taxa, such as *Dieffenbachia* spp., these raphides are found in specialized ovate-shaped cells called idioblasts. Characterized by openings at both termini, idioblast cells function as encapsulating units. Upon the application of mechanical force, these idioblasts facilitate the expulsion of raphides, propelling them over a distance equivalent to the length of two to three cells. The resulting mechanical energy, facilitated by the distinctive architecture of the calcium oxalate crystals, causes localized cellular damage. Notably, there is evidence that suggests the potential additional presence of toxic components within these idioblasts [24]. This leads to similar toxicological effects as *Colocasia* [22,23,25]. Our results confirm these, showing similar symptoms in cases of *Dieffenbachia* and *Colocasia* exposure.

Both *Colocasia* and *Dieffenbachia* are common indoor and outdoor ornamental plants in the study region. This highlights the importance of parental awareness and education on the potential poisonous hazards of houseplants, especially those that are easily accessible to children.

*Ricinus communis*, commonly known as “castor bean”, is a highly toxic plant of significant commercial importance as the primary source of castor oil. Historically, castor oil has been used as a laxative and as a lubricant for industrial machinery. Notably, all components of the plant are toxic, with the seeds being particularly potent in their toxic effects. *Ricinus communis* reaches a height of 1.5 to 4.5 m at maturity and has distinctive palmate leaves. The seeds are clustered near the top of the plant and are enclosed in a spiny husk [1].

The toxicity of *Ricinus communis* is primarily attributed to the presence of toxalbumins, a class of proteins that selectively bind to carbohydrates. Of particular interest is the toxin known as ricin, found in the bean pulp of the plant, which is inactivated under heated conditions during oil extraction; therefore, castor oil or derived products are not expected to be toxic. This toxin has been shown to produce a range of toxicological effects when administered parenterally to animals. These effects include an increase in cardiac output, induction of cardiac hemorrhage and necrosis, provocation of coronary vasospasm, and impairment of both systolic and diastolic cardiac function [22].

In addition, its seeds contain a hemagglutinin component. Interestingly, oral administration of this hemagglutinin does not appear to induce hemolysis, as observed in previous studies. The lethality of poisoning depends on the route of exposure, with inhalation and injection being the most lethal routes. Ingestion is responsible for gastrointestinal symptoms such as nausea, vomiting, and bloody diarrhea, which can lead to severe dehydration and multiple organ damage, and in severe cases, death [24,26].

Five cases of *Ricinus* poisoning were identified in our study, with all patients presenting gastrointestinal symptoms (nausea, vomiting, abdominal pain, and choleriform diarrhea). All of these were classified as moderate cases of poisoning with a Poisoning Severity Score of 2, as documented in other studies [22,27].

Besides *Colocasia*, *Dieffenbachia*, and *Ricinus*, three other highly toxic plants were responsible for acute cases of exposure in our study group: *Datura stramonium*, *Aconitum*, and *Atropa belladonna*.

*Datura stramonium*, commonly known as “jimson weed” or “thorn apple”, is a well-known recreational plant that has been also used for centuries for various medicinal purposes. It contains several toxic compounds, including atropine, scopolamine, and hyoscyamine, which activate the anticholinergic system. The seeds of *Datura stramonium* are particularly rich in these toxins, which, when ingested, cause a range of symptoms similar to atropine poisoning. These include dry mouth and skin, extreme thirst, mydriasis with blurred vision, urinary retention, tachycardia, confusion, agitation, hallucinations, and a loss of consciousness [28]. The severity of symptoms may vary depending on the dose ingested, and severe cases may require hospitalization and supportive care, including intravenous fluids and mechanical ventilation [29,30,31]. There were three cases of *Datura stramonium* poisoning in our study group, which consisted of three male adolescents with acute intentional ingestion. Their medical histories revealed that they had ingested Datura seeds for recreational purposes. The symptoms observed in these patients were delirium, confusion, visual hallucinations, mydriasis, dysarthria, dizziness, and dry mouth, all in accordance with the literature. These cases of poisoning were classified as moderate (PSS = 2), and treatment was symptomatic.

The genus *Aconitum* (Ranunculaceae) is represented by a variety of species rich in alkaloids such as aconitine, which are responsible for its cardiotoxicity, neurotoxicity, and gastrointestinal toxicity. Its toxicity is explained by the interaction of alkaloids with Na+ channels, causing cell depolarization and permanent activation of these channels. Sodium overload of cardiac cells is also responsible for severe ventricular arrhythmias, which are the main cause of death in this form of poisoning [32]. The two patients intoxicated with *Aconitum* identified in our study did not present any cardiological signs or symptoms, but they did suffer from gastrointestinal effects, which are not uncommon in the literature on this type of poisoning [33].

*Atropa belladonna*, also known as “deadly nightshade”, is a large perennial herbaceous plant native to Europe. Although sometimes cultivated as ornamental, this plant is notorious for its highly toxic properties. Its cherry-like berries, which are dark purple/black and contain numerous seeds, are the most poisonous part of the plant. *Atropa belladonna* contains tropane alkaloids, including atropine, scopolamine, and hyoscyamine, which have medicinal uses as anticholinergics. However, in high doses, these alkaloids can be extremely toxic, resulting in a potentially life-threatening anticholinergic toxidrome [34]. The two cases of *Atropa belladonna* poisoning found in our study that presented exclusively with digestive symptoms were of moderate severity (PSS = 2) and had a favorable outcome.

Overall, the clinical presentation varied according to the type of plant, with gastrointestinal symptoms being the most common. These findings are consistent with previous studies which suggest that intestinal disturbances are most commonly encountered in plant poisoning [16,35].

Plant exposure seldom causes severe symptoms, with less than one-third of the children admitted to hospital being symptomatic [16,35]. In our study, the assessment of symptom severity was performed using the Poisoning Severity Score (PSS), which showed that 28% of patients were asymptomatic, and the rest showed mild symptoms.

Length of hospitalization was short in our group study, with the median hospitalization being 2 days; 65 (91.5%) of the patients were hospitalized for 2 days or less. This is consistent with the literature, which indicates a median length of hospitalization between 1 and 2 days, even in severe cases [9,17].

In this study, which analyzed hospitalized patients, length of hospitalization was a good measure of severity and, together with PSS, showed that plant exposure did not represent a major danger in the pediatric population. However, preventive measures, the accurate identification of causative plant species, the early recognition of signs and symptoms, and the prompt initiation of appropriate treatment must remain a target for specialists.

Treatment was symptomatic in all cases and consisted of antiemetics, water–electrolyte re-equilibration, or antiallergic treatment (antihistamine drugs and/or corticoids). These findings suggest that careful management and supportive care can effectively address the symptoms of toxic exposure without the need for specific antidote treatment in most cases. All patients were discharged without sequelae.

The main limitation of this study is that the sample size was too small, which is explained by the fact that only patients admitted to hospital were included, since asymptomatic or mildly symptomatic patients do not access hospital facilities, which means that these cases were underreported. This sample could have been more representative if more medical centers were included; however, our center provides the most important toxicological emergencies from a wide area of our country. Also, this study has other several limitations. The interpretation of our data was limited by the retrospective analysis. Another important limitation is related to the modality of plant identification, which in most cases was based only on the reports of the patient or their family.

However, this study provides valuable information that could potentially improve the approach to plant exposure in children and adolescents. Although there are only a few cases, the study describes the clinical and paraclinical characteristics of 30 different species of plants. This research also underscores the importance of accurate identification of causative plant species, the early recognition of the clinical picture, and the initiation of correct treatment strategies in these situations. Furthermore, the data from this study contribute to supplementing pre-existing information on plant exposure in Central and Eastern Europe.

## 5. Conclusions

The number of pediatric cases of plant exposure requiring hospitalization was low, with most cases classified as mild. Most of the cases were unintentional exposures, in children under 5 years of age. Occasionally, intentional poisonings were reported in adolescents who use plants for recreational purposes.

The characteristic route of plant exposure was the digestive tract and the most common symptoms were gastrointestinal, with vomiting being predominant.

*Colocasia, Dieffenbachia*, and *Ricinus* were the plants most commonly involved, with the latter two being classified as highly toxic plants.

In all the cases, treatment consisted of supportive and symptomatic measures. 

Even though it may not appear to be a major health problem, educating the public on how to prevent, identify, and recognize early symptoms should remain a priority for toxicologists.

## Figures and Tables

**Table 1:** Demographic data—the most affected age group was under 5 years old, and the most common reason was accidental exposure.

	Age			Gender		Area		Reason		
Category	≤5 Years	6–12 Years	13–18 Years	Male	Female	Urban	Rural	Intentional	Unintentional	Other
Number	51	14	6	32	39	40	31	4	66	1
Percentage	71%	19%	8.00%	45%	54%	56%	43%	5%	92%	1%

**Table 2:** Clinical picture and poisoning severity score related to plant species in acute plant poisoning cases—Clinical picture depends on the plant species, with symptoms being mild or moderate in most cases.

Type of Plant	Number of Cases	Signs and Symptoms	Biological Findings	Poisoning Severity Score (PSS)	Number of Cases
*Colocasia* (Elephant’s ear or taro)	13 (18.3%)	excessive salivation, vomiting, Quincke’s edema, local hyperemia, nausea, itchy skin, dysphagia, dizziness, hallucinations, oral cavity injury, cough	none	PSS-0	0
				PSS-1	9
				PSS-2	4
				PSS-3	0
				PSS-4	0
*Dieffenbachia* (Dumb canes)	7 (9.9%)	local hyperemia, vomiting, excessive salivation, oral cavity injury	alkalosis	PSS-0	1
				PSS-1	6
				PSS-2	0
				PSS-3	0
				PSS-4	0
*Ricinus communis* (Ricinus)	5 (7%)	vomiting, nausea, abdominal pain, oral cavity injury, sleepiness	hyperglycemia	PSS-0	1
				PSS-1	0
				PSS-2	4
				PSS-3	0
				PSS-4	0
*Atropa belladonna* (Deadly nightshade)	4 (5.6%)	local hyperemia, vomiting, abdominal pain, oral cavity injury, cough, restless	hyperglycemia	PSS-0	1
				PSS-1	2
				PSS-2	1
				PSS-3	0
				PSS-4	0
*Phytolacca decandra* (Dragon berries)	4 (5.6%)	oral cavity injury, vomiting, abdominal pain, cough	none	PSS-0	2
				PSS-1	2
				PSS-2	0
				PSS-3	0
				PSS-4	0
*Datura stramonium* (Thorn apple)	3 (4.2%)	dizziness, hallucinations, restless, balance disorder, conjunctival hyperemia, bilateral mydriasis, sore mouth	hyperglycemia	PSS-0	0
				PSS-1	0
				PSS-2	3
				PSS-3	0
				PSS-4	0
*Sambucus* spp. (Black elderberry)	3 (4.2%)	vomiting, diarrhea, oral cavity injury	none	PSS-0	2
				PSS-1	1
				PSS-2	0
				PSS-3	0
				PSS-4	0
*Aconitum* (Wolf’s bane)	2 (2.8%)	nausea, vomiting, dysphagia, oral cavity injury	hypoglycemia	PSS-0	0
				PSS-1	0
				PSS-2	2
				PSS-3	0
				PSS-4	0
*Hedera* (Ivy)	2 (2.8%)	diarrhea, oral cavity injury, cough	acidosis	PSS-0	1
				PSS-1	1
				PSS-2	0
				PSS-3	0
				PSS-4	0
*Hevea brasiliensis* (Rubber tree)	2 (2.8%)	Quincke’s edema, local hyperemia, excessive salivation, oral cavity injury	none	PSS-0	0
				PSS-1	2
				PSS-2	0
				PSS-3	0
				PSS-4	0
*Nerium oleander* (Oleander)	2 (2.8%)	vomiting, diarrhea, oral cavity injury, sleepiness	hyperglycemia	PSS-0	2
				PSS-1	0
				PSS-2	0
				PSS-3	0
				PSS-4	0
*Solanum capsicum* (Jerusalem cherry)	2 (2.8%)	none	none	PSS-0	1
				PSS-1	1
				PSS-2	0
				PSS-3	0
				PSS-4	0
*Taxus baccata* (English yew)	2 (2.8%)	oral cavity injury, cough	none	PSS-0	2
				PSS-1	0
				PSS-2	0
				PSS-3	0
				PSS-4	0
*Viscum album* (Mistletoe)	2 (2.8%)	induced vomiting	acidosis	PSS-0	2
				PSS-1	0
				PSS-2	0
				PSS-3	0
				PSS-4	0
Unidentified plant	2 (2.8%)	local hyperemia, nausea, vomiting, abdominal pain, excessive salivation, dysphagia, oral cavity injury, cough, sleepiness	none	PSS-0	0
				PSS-1	1
				PSS-2	1
				PSS-3	0
				PSS-4	0
*Amaranthus caudatus* (Love-lies-bleeding)	1 (1.4%)	none	none	PSS-0	1
				PSS-1	0
				PSS-2	0
				PSS-3	0
				PSS-4	0
*Arum alpinum* (Mountain arum)	1 (1.4%)	oral cavity injury	none	PSS-0	0
				PSS-1	1
				PSS-2	0
				PSS-3	0
				PSS-4	0
*Conium maculatum* (Hemlock)	1 (1.4%)	excessive salivation	none	PSS-0	0
				PSS-1	1
				PSS-2	0
				PSS-3	0
				PSS-4	0
*Hyacinthus orientalis* (Hyacinth)	1 (1.4%)	nausea, diarrhea, abdominal pain	hyperglycemia	PSS-0	0
				PSS-1	1
				PSS-2	0
				PSS-3	0
				PSS-4	0
*Laburnum anagyroides* (Golden chain)	1 (1.4%)	oral cavity injury, balance disorder, lower-limb hypotonia	acidosis, hyperglycemia	PSS-0	0
				PSS-1	1
				PSS-2	0
				PSS-3	0
				PSS-4	0
*Litchi chinensis* Lychee)	1 (1.4%)	none	hyperglycemia	PSS-0	1
				PSS-1	0
				PSS-2	0
				PSS-3	0
				PSS-4	0
*Platanus* (Platanus)	1 (1.4%)	vomiting, oral cavity injury	none	PSS-0	0
				PSS-1	1
				PSS-2	0
				PSS-3	0
				PSS-4	0
*Pyracantha coccinea* (Scarlet firethorn)	1 (1.4%)	nausea, vomiting, cough, sleepiness	none	PSS-0	0
				PSS-1	1
				PSS-2	0
				PSS-3	0
				PSS-4	0
*Solanum dulcamara* (Bitter nightshade)	1 (1.4%)	oral cavity injury	none	PSS-0	1
				PSS-1	0
				PSS-2	0
				PSS-3	0
				PSS-4	0
*Solanum nigrum* (European black nightshade)	1 (1.4%)	oral cavity injury	none	PSS-0	1
				PSS-1	0
				PSS-2	0
				PSS-3	0
				PSS-4	0
*Spathiphyllum wallisii* (Peace lily)	1 (1.4%)	vomiting	none	PSS-0	0
				PSS-1	1
				PSS-2	0
				PSS-3	0
				PSS-4	0
*Strychnos nux vomica* (Nux vomica)	1 (1.4%)	none	none	PSS-0	1
				PSS-1	0
				PSS-2	0
				PSS-3	0
				PSS-4	0
*Symphoricarpos albus* (Snowberry)	1 (1.4%)	local hyperemia	none	PSS-0	0
				PSS-1	1
				PSS-2	0
				PSS-3	0
				PSS-4	0
*Syngonium podophyllum* (Arrowhead vine)	1 (1.4%)	dysphagia, oral cavity injury	none	PSS-0	0
				PSS-1	1
				PSS-2	0
				PSS-3	0
				PSS-4	0
*Wisteria* spp. (Wisteria)	1 (1.4%)	vomiting, oral cavity injury, cough	none	PSS-0	0
				PSS-1	1
				PSS-2	0
				PSS-3	0
				PSS-4	0
*Zamioculcas zamiifolia* (ZZ plant)	1 (1.4%)	acute urticaria, oral cavity injury	hyperglycemia	PSS-0	0
				PSS-1	1
				PSS-2	0
				PSS-3	0
				PSS-4	0

## Data Availability

The data presented in this study are available on request from the corresponding author. The data are not publicly available to ensure privacy and confidentiality as they have not been fully anonymized.
